# Multilevel Societies in New World Primates? Flexibility May Characterize the Organization of Peruvian Red Uakaris (*Cacajao calvus ucayalii*)

**DOI:** 10.1007/s10764-012-9603-6

**Published:** 2012-05-26

**Authors:** Mark Bowler, Christoph Knogge, Eckhard W. Heymann, Dietmar Zinner

**Affiliations:** 1 School of Psychology, University of St. Andrews, KY16 9JP St. Andrews, UK; 2 Behavioral Ecology & Sociobiology Unit, German Primate Center (DPZ), 37077 Göttingen, Germany; 3 IPÊ – Institute for Ecological Research, Caixa Postal 47-12960-000 Nazaré Paulista, Brazil; 4 Behavioral Ecology & Sociobiology Unit, German Primate Center (DPZ), 37077 Göttingen, Germany; 5 Cognitive Ethology Laboratory, German Primate Center (DPZ), 37077 Göttingen, Germany

**Keywords:** Breeding system, Mating system, One-male unit, Pitheciine, Social structure

## Abstract

Researchers have described multilevel societies with one-male, multifemale units (OMUs) forming within a larger group in several catarrhine species, but not in platyrhines. OMUs in multilevel societies are associated with extremely large group sizes, often with >100 individuals, and the only platyrhine genus that forms groups of this size is *Cacajao*. We review available evidence for multilevel organization and the formation of OMUs in groups of *Cacajao*, and test predictions for the frequency distribution patterns of male–male and male–female interindividual distances within groups of red-faced uakaris (*Cacajao calvus ucayalii*), comparing year-round data with those collected at the peak of the breeding season, when group cohesion may be more pronounced. Groups of *Cacajao* fission and fuse, forming subgroup sizes at frequencies consistent with an OMU organization. In *Cacajao calvus ucayalii* and *Cacajao calvus calvus*, bachelor groups are also observed, a characteristic of several catarrhine species that form OMUs. However, researchers have observed both multimale–multifemale groups and groups with a single male and multiple females in *Cacajao calvus*. The frequency distributions of interindividual distances for male–male and male–female dyads are consistent with an OMU-based organization, but alternative interpretations of these data are possible. The distribution of interindividual distances collected during the peak breeding season differed from those collected year-round, indicating seasonal changes in the spatial organization of *Cacajao calvus ucayalii*. We suggest a high degree of flexibility may characterize the social organization of *Cacajao calvus ucayalii*, which may form OMUs under certain conditions. Further studies with identifiable individuals, thus far not possible in *Cacajao*, are required to confirm the social organization.

## Introduction

Researchers have described multilevel societies in several catarrhine species, e.g., hamadryas baboons (*Papio hamadryas*), geladas (*Theropithecus gelada*), and snub-nosed monkeys (*Rhinopithecus* spp.) (Grueter *et al.* 2012b). These species are characterized by large groups or troops that are composed of smaller one-male–several-female groups or one-male units (OMUs). In some cases, additional intermediate levels between OMUs and troops are found, e.g., in hamadryas baboons, in which OMUs form clans and clans form bands (Abegglen 1984; Schreier and Swedell 2009). Multilevel organization in catarrhines has been related to exceptionally large group or aggregation sizes, in some cases several hundred individuals (Grueter *et al.* 2012b).

Multilevel societies with OMUs have evolved independently in two major catarrhine clades with different ancestral social organizations: multimale–multifemale (mm–mf) groups in the papionins, and most likely single OMUs in the Colobinae. The first efforts to model the evolution of social organization analyzed the relationship between ecology and social structures (Clutton-Brock and Harvey 1976; Crook and Gartlan 1966), later incorporating further codependent factors such as male and female strategies (Wrangham 1979, 1980). This eventually resulted in socioecological models relating social organization to differing food availability and distribution, predation risk, and risk of infanticide by males (Barton 2000; Fairbanks and Bird 1978; Sterck *et al*. 1997; van Schaik 1983, 1989, 1996; van Schaik and Kappeler 1997). Bachelor groups or all-male units (AMUs) are characteristic of some species that form OMUs within larger groups such as snub-nosed monkeys and geladas (Grueter and van Schaik 2010; Grueter and Zinner 2004; Kirkpatrick and Grueter 2010), but not hamadryas baboons (Pines *et al*. 2011; Swedell *et al*. 2011). The threat imposed by bachelor groups is considered to be a driving factor leading to the formation of large groups consisting of OMUs in several species: zebras (*Equus quagga*: Rubenstein and Hack 2004), snub-nosed monkeys, and geladas (Grueter and van Schaik 2010; Grueter *et al.* 2012a).

Although sociogeological factors will be important in modeling the evolution of primate social systems, these systems represent most likely a combination of adaptations to present-day environment and phylogenetic inertia (Chapman and Rothman 2009). However, some mating systems (as one component of the social system *sensu* Kappeler and van Schaik 2002) should be less constrained by phylogenetic inertia than others (Pope 2000), and sociospatial organization and social relationships seem to be even less constrained and more flexible in adaption to environmental and social conditions. They may vary within species, e.g., in *Semnopithecus* (Jay 1965; Sugiyama 1964) or between closely related species living under different conditions (Papionini: Dunbar 1986; Kummer 1968; Stammbach 1987; guenons: Cords 1988; capuchins: Izar *et al*. 2011). The prevalence of such social systems in unrelated catarrhines with extremely large group sizes allows us to hypothesize that a similar organization might occur within other primates that live in similarly large groups, which must experience similar selection pressures on their social system.

Multilevel systems have never been demonstrated in platyrrhines, perhaps because group sizes generally tend to be smaller than in catarrhines (Kappeler and Heymann 1996). However, large groups, comparable in size to those of OMU-forming catarrhines, with low group cohesion, are typically found in uakaris, genus *Cacajao*. The extremely large group sizes (for platyrrhines) seen in *Cacajao calvus ucayalii* make them the most likely candidate within platyrrhines in which to find a multilevel organization. Black-faced uakaris, traditionally considered as a single species, *Cacajao melanocephalus*, are now split into three species: *Cacajao melanocephalus*, *Cacajao hosomi*, and *Cacajao ayresi* (Boubli *et al*. 2008) usually occur in groups of ≥100, and most groups studied show a fission–fussion grouping pattern (*sensu* Aureli *et al*. 2008; Chapman *et al*. 1993), with low cohesion and short-term changes in group sizes (Barnett and Brandon-Jones 1997; Barnett and da Cunha 1991; Bezerra *et al*. 2010; Defler 1999). However, a group of 70 *Cacajao hosomi* in caatinga forests did not fission, but spread out widely during travel (Boubli 1997, 1999). Within the red-faced uakaris (*Cacajao calvus* ssp.: Hershkovitz 1987), groups of 45–48 *Cacajao calvus calvus* fissioned regularly, often spending several days apart (Ayres 1986,1989). Researchers have observed Peruvian red uakaris (*Cacajao calvus ucayalii*) in groups of ≥200 individuals (Aquino 1998; Bowler and Bodmer 2009, 2011), and fission–fusion also appears to be the norm in this subspecies. The question here is whether uakaris cleave in highly predictable ways according to established membership in social subunits, e.g., OMUs, often kin-based, within larger units or whether fissioning occurs in a more random fashion with resulting subunits being highly variable in size and membership.

Data on the social behavior of *Cacajao* are sparse, but here we review the available evidence for patterns that might indicate a multilevel organization in uakari groups, and the formation of OMUs, also considering evidence from other pitheciine primates, especially the very closely related *Chioropotes*. In doing so, we hope to point to gaps in our knowledge and guide future research. We also present new preliminary data to test whether the observed spatial arrangement of wild groups of *Cacajao calvus ucayalii* fits predictions made for groups forming OMUs for which we would expect the frequency distributions of male–male (m–m) and male–female (m–f) interindividual distances to be bimodal, with each peak representing the distribution of interindividual distances for males in OMUs and males in AMUs. We also compare the frequency distributions for data collected year-round with data collected at the peak of the breeding season, when we predict AMUs will be closer to OMUs.

## Review of Existing Data

### Multilevel Societies in Uakaris

The reported low cohesion within groups of red uakaris implies multilevel organization. Heymann (1992) hypothesized that the social organization of *Cacajao calvus ucayalii* comprises three levels: the troop (*ca*. 50 to >100 individuals), which is composed of several groups (*ca*. 25–50 individuals), which in turn comprises several foraging units (up to *ca*. 10 individuals). The distribution of group sizes observed within a local population of >200 individuals in the Lago Preto Conservation Concession (Bowler and Bodmer 2009) provides limited support for the existence of these three tiers. The average number of uakaris associating was 43.5 ± 24.1 individuals (mean ± SD, *N* = 759), but group sizes ranged from 1 to >150 individuals (Bowler and Bodmer 2009). However, the same patterns could be created by a simpler two-tiered organization in which units aggregate in nonconsistent ways. Because individuals were not identified during the study it was not possible to measure the consistency of group membership. In *Cacajao calvus calvus*, only groups of up to 48 have been observed; this intensively studied group frequently divided into smaller units, but it was isolated from other groups with which it could have formed larger troops (Ayres 1986).

### Uakari Social Organization and Mating System

The first study of the social behavior of *Cacajao* was on a captive group of *Cacajao calvus ucayalii*, in which mating was said to be promiscuous, implying a mm–mf social organization and mating system (Fontaine 1981). Observations of mating in wild uakaris have been of pairs that were not in the vicinity of other individuals (*N* = 6, Bowler and Bodmer 2009; *N* = 1, C. Knogge *unpubl. data*). These could be similar to the consort pairs found in mm–mf groups of baboons (Seyfarth 1978). Ayres (1986) made some limited observations on the social behavior of *calvus*, noting the temporary formation of pairs during the mating season, which were harassed by bachelor male groups of up to eight or nine individuals. On the basis of these observations, Ayres (1986) suggested a single-male mating system for uakaris, which implies an OMU social organization that could include pairs or single-male, multifemale groups. Researchers have also observed bachelor groups (AMUs) in *ucayalii* (Bowler and Bodmer 2009), but not in black-faced uakaris, despite increasing numbers of studies (Barnett *et al*. 2005; Bezerra *et al.* 2010; Defler 1999). Gregory (2011) observed AMUs in Guianan bearded sakis (*Chiropotes sagulatus*), but most other groups of bearded sakis did not form male-only groups (Ayres 1989; Silva and Ferrari 2009; Veiga and Silva 2005; Veiga *et al*. 2006), although this might be due to a lower frequency of group fissions than in *Cacajao*.

### Spatial Arrangement of Age–Sex Classes

The spatial configuration and composition of the smallest foraging units can be used as indicators for characterizing the uakaris’ lowest organizational level, deriving evidence for the mating system and the organizational structure of the multilevel society. In an OMU organization, we would expect to see females as the nearest neighbors of breeding adult males more often than other males. In an mm–mf mating system, we could find that males are the most common nearest neighbors of breeding adult males, as found in *Ateles* and *Brachyteles* (Strier *et al*. 2002; Symington 1990), or male–male associations might not occur, as in *Cebus* (Fragaszy *et al*. 2004) or *Lagothrix* (Di Fiore and Fleisher 2005).

Although M. Bowler frequently observed and recorded on video multiple males traveling in close proximity with pregnant females and those with young infants, nearest neighbor studies on *ucayalii* at Lago Preto were hindered by the difficulty of identifying individuals within the local population of >200 individuals (Bowler and Bodmer 2009), and the spatial organization was recorded only for age–sex classes. Adult males were recorded most commonly as the nearest neighbors of adult and subadult males, and adult females most commonly as the nearest neighbors of adult females. This is compatible with the mm–mf model, but no distinction could be made between males in bachelor units and males associating with adult females, and an OMU system could produce the same result through proximity between bachelor males. At Lago Preto, adult and subadult males were more often >30 m from their nearest neighbors than all other age–sex classes (Bowler and Bodmer 2009), indicating that groups were accompanied by peripheral males. There is no information on the spatial arrangement of age–sex classes from any other population of *Cacajao*.

### Male–Male Affiliation and Coalitions Within mm–mf Groups

Greater levels of m–m affiliation may be possible between males in mm–mf groups than between males from different OMUs. This is a consequence of the smaller m–m distances in mm–mf groups, although there is also the possibility that males could increase their reproductive success through coalitions facilitated through m–m affiliation. Although m–m affiliation, including frequent grooming and spatial proximity, is not always seen within mm–mf primate groups, e.g. *Cebus* (Fragaszy *et al*. 2004), *Lagothrix* (Di Fiore and Fleisher 2005), and *Papio ursinus* (Swedell 2011), several species do have affiliative males, e.g., *Brachyteles* (Strier *et al*. 2002), and *Ateles* (Symington 1990). A high level of adult m–m affiliation appears the norm in *Chiropotes* (Gregory 2011; Veiga and Silva 2005), and also occurs in adult male *ucayalii* (Bowler and Bodmer 2009). Affiliation between males is not in itself evidence for an mm–mf mating system because affiliative behavior also occurs within bachelor groups in species that form OMUs, e.g. geladas (Dunbar 1986). Even in hamadryas baboons in which bachelor groups are absent, solitary males may groom each other and spend the night in close proximity on the sleeping cliff (Pines *et al* 2011; Swedell and Plummer 2012).

In addition to m–m grooming dyads, male uakaris at Lago Preto also engaged in aggressive chasing behaviors between cooperating m–m coalitions (*sensu* van Schaik *et al*. 2006) of up to eight or more individuals (Bowler and Bodmer 2009), but it was not possible to determine whether breeding males or only males in bachelor groups formed these coalitions (Bowler and Bodmer 2009).

## New Data: Methods

### Study Site

The Lago Preto Conservation Concession (LPCC: S04°27.5′ W071°45.9′) is a 9926-ha public–private reserve, 175 km southeast of Iquitos, Peru on the border of Brazil. Within the Concession, the Lago Preto study area includes 1400 ha of nonflooding *terra firme* forest, 500 ha of white-water *várzea* forest that floods each year with silt-laden water between November and May, and 300 ha of *aguajal* palm swamps, permanently waterlogged forest dominated by *Mauritia flexuosa* palms. Groups of ≤150 uakaris occurred throughout the study area, typically fissioning and fusing through the day (Bowler and Bodmer 2009).

### Behavioral Methods

M. Bowler followed groups of *Cacajao calvus ucayalii* for 945 h between April 2003 and July 2005, collecting data in all months except February 2004, April 2005, and May 2005. At 10-min intervals, M. Bowler randomly selected one focal individual (from all age–sex classes; Bowler and Bodmer 2009) and estimated interindividual distances between the focal individual and all its visible and identifiable neighbors within 30 m. Low visibility meant that we could not always identify the age–sex class of both focal individuals and their neighbors. We used interindividual distances for dyads of m–m and m–f adult animals (*N* = 168) for analysis. Bowler and Bodmer (2009) analyzed the age–sex classes of the nearest neighbors of focal individuals using these data, but did not use interindividual distances.

C. Knogge collected data at LLPC, locating and following the same groups habituated by M. Bowler (Bowler and Bodmer 2009), over a period of 14 d between June 17 and July 11, 2006, within the mating season for red uakaris (Bowler *et al*. in press; C. K*. this study*). During 30 h of contact, C. Knogge estimated interindividual distances of all visible individuals during 3-min scan samples at 10-min intervals, whenever possible indentifying sex and age class (adult, subadult, young) of the subjects. C. Knogge recorded 184 interindividual distances between categorized individuals (m–m, m–f, female–female [f–f]) and 79 dyads in which only one dyad partner could not be categorized.

### Data Analysis

**Fig. 1:**
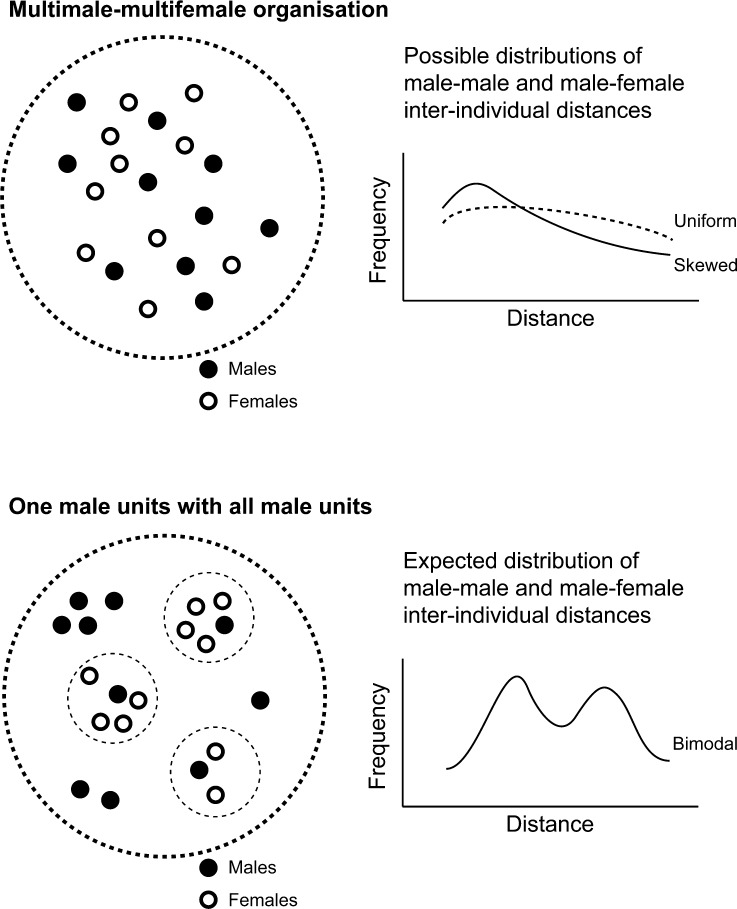
Expected distribution patterns for nearest neighbor distances in multimale–multifemale and one-male units in *Cacajao calvus ucayalii*.

We analyzed year-round data collected by M. Bowler and mating season data collected by C. Knogge separately. We compared the observed frequency distribution patterns of interindividual distances with expected patterns. For uakari groups organized in OMU and AMUs we expected a pattern of m–m distances with two peaks, reflecting the two categories of males, with closer distances between males of AMUs and longer distances between single males in OMUs. For m–f distances, we expected two peaks: one reflecting distances between the OMU male and his guarded females and the other resulting from greater distances between satellite males in AMUs and the females in OMUs (Fig. 1). For an mm–mf organization we expected a uniform or skewed distribution of interindividual distance frequencies between m–f and m–m dyads. We had no clear predictions for the distribution f–f interindividual distances.

## New Data: Results

### Year-Round Interindividual Distances

**Fig. 2:**
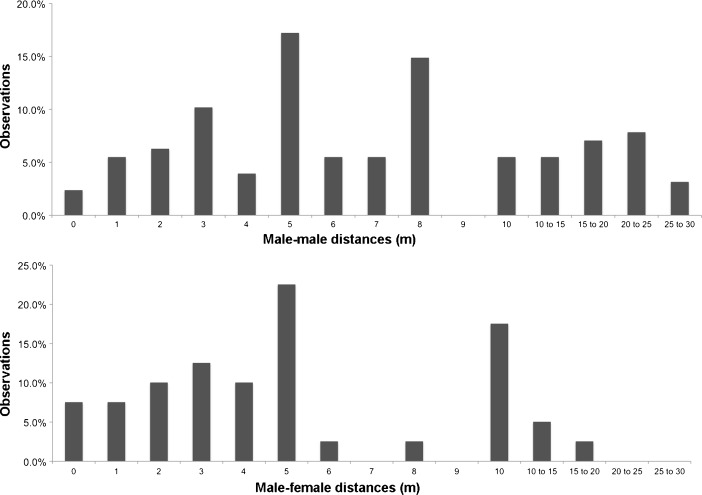
Nearest neighbor distance distribution patterns for male–female and male–male dyads in *Cacajao calvus ucayalii* recorded by M. Bowler from year-round data, April 2003 to July 2005.

During the study by M. Bowler between April 2003 and July 2005 the frequency distribution of the interindividual distances for m–f and m–f associations appear to most closely fit the expected distributions for OMUs (Fig. 2). In contrast, anecdotal observations suggested that adult females and their offspring were often accompanied by more than one adult male.

### Breeding Season Interindividual Distances

**Fig. 3:**
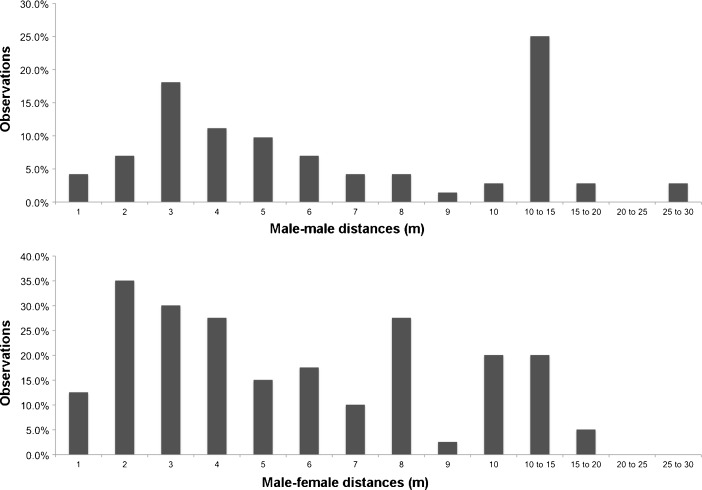
Nearest neighbor distance distribution patterns for male–female and male–male dyads in *Cacajao calvus ucayalii* recorded by C. Knogge during the breeding season, June–July 2006.

**Fig. 4:**
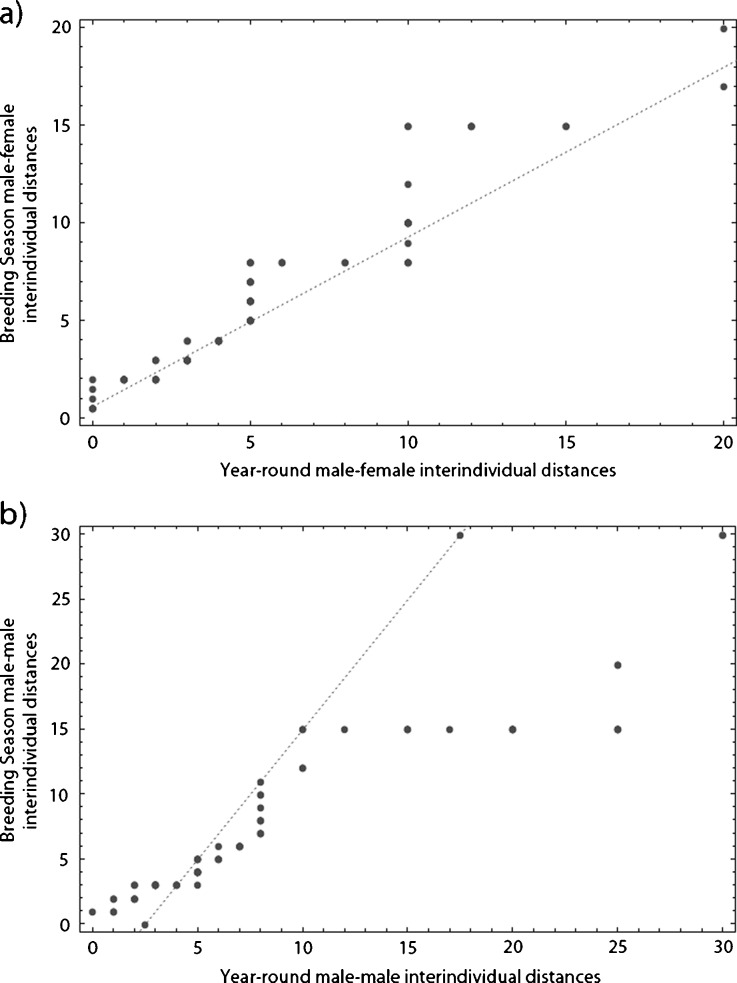
Quantile–quantile plots of the nearest neighbor distances of *Cacajao calvus ucayalii* during the breeding season and year-round for male–female (**a**) and male–male (**b**) dyads. Deviation from a linear relationship indicates differing distribution patterns for the frequency of recorded nearest neighbour distances collected during the breeding season.

During the study by C. Knogge in June and July 2006, red uakari subgroups, defined as all the individuals detected in the area by the researcher, were composed of 15–33 individuals. The frequency distributions of the interindividual distances for m–m and f–m appear to most closely fit the expected distributions for OMUs (Fig. 3). Anecdotal observations suggested that the spatial configuration and composition of the foraging units was based on small OMUs with surrounding AMUs (five males or fewer). Typically the small units comprised two adult females and offspring, two subadults, and one adult male. In the breeding season, the frequency distributions of m–f interindividual distances were similar to those in the year-round data (Fig. 4a). However, the distributions of m–m interindividual distances in the breeding season differed from those in the year-round data (Fig. 4b). The second peak of m–m interindividual distances was at shorter distances in the breeding season than in year-round data (Fig. 3).

## Discussion

The few studies on uakaris that have been conducted provide limited evidence for the social organization of *Cacajao calvus*. The lack of cohesion in groups suggests that there might be multiple levels to their societies (Heymann 1992), but the consistency of membership of subgroups has not been tested. Groups containing several adult females and multiple adult males that Bowler and Bowler (2009) observed suggest an mm–mf organization, but a lack of individual identification meant that this could not be confirmed. AMUs occurred in both *Cacajao calvus calvus* and *Cacajao calvus ucayalii* (Ayres 1986; Bowler and Bodmer 2009) and are characteristic of many primate societies in which OMUs form, but are not diagnostic of an OMU social organization.

Care must be taken in interpreting the results of the behavioral studies presented here; although the graphs fit the predictions made for groups containing OMUs, other interpretations of the data are possible. The second peaks in the frequencies of inter-individual distances could result from the peripheral males that occur at Lago Preto (Bowler and Bodmer 2009). Although the weight of evidence appears to favour a mm–mf system for *Cacajao calvus*, researchers have observed units apparently containing a single adult male in separate studies by Ayres (1986), Bowler and Bodmer (2009), and C. Knogge (*this study*). Therefore, we cannot rule out the formation of OMUs. One interesting difference between the year-round and mating season data collected by C. Knogge and M. Bowler was that the second peak frequency of interindividual distances for m–m dyads was at much shorter distances in the mating season. Although interobserver reliability could not be tested, the difference is too large to be accounted for by observers’ differences in distance estimates. The discrepant pattern could be a result of bachelor male groups or peripheral males coming closer to OMUs or mm–mf subgroups during the breeding season.

### Seasonal Flexibility in Uakari Social Organization

We suggest a high degree of flexibility may characterize the social organization of *Cacajao calvus*, which may form OMUs under certain conditions. The discrepancy between results and observations made during long- and short-term studies at Lago Preto (Bowler and Bodmer 2009; *this study*) could lie in the highly seasonal breeding and feeding patterns found in *Cacajao calvus*; seasonal and synchronized breeding occurs in *C. calvus* (Ayres 1986; Bowler and Bodmer 2009; Bowler *et al*. in press), and strong seasonal fluctuation in the abundance of key resources (Ayres 1986; Bowler and Bodmer 2011) creates great temporal variation in the selection pressures on uakari social systems. Ecological pressures will determine the cohesion of females in groups, while the pressures on males to go where the females are will vary temporally with the need to gain access to mates during the breeding season and defend their offspring against potential infanticide when infants are small (van Schaik and Kappeler 1997). It is possible that uakari group composition is so flexible that both OMU and mm–mf foraging or breeding units can occur through the year depending on resource distribution and the number of ovulating females or young infants present. Researchers have also recorded flexibility and seasonal variation in social systems in mandrills (*Mandrillus sphinx*), in which mature males are most likely present only seasonally (Abernethy *et al*. 2002), in patas monkeys (*Erythrocebus patas*), red-tailed monkeys (*Cercopithecus ascanius*), and blue monkeys (*Cercopithecus mitis*), in which researchers have observed seasonal influxes of males into single-male, multi-female groups (Carlson and Isbell 2001; Chism and Rogers 1997; Chism and Rowell 1986), and in baboons, in which strong f–f bonds and cliques form more frequently in lean periods than in times of plenty (Henzi *et al*. 2009).

### Evolution of the Uakari Social System

**Fig. 5:**
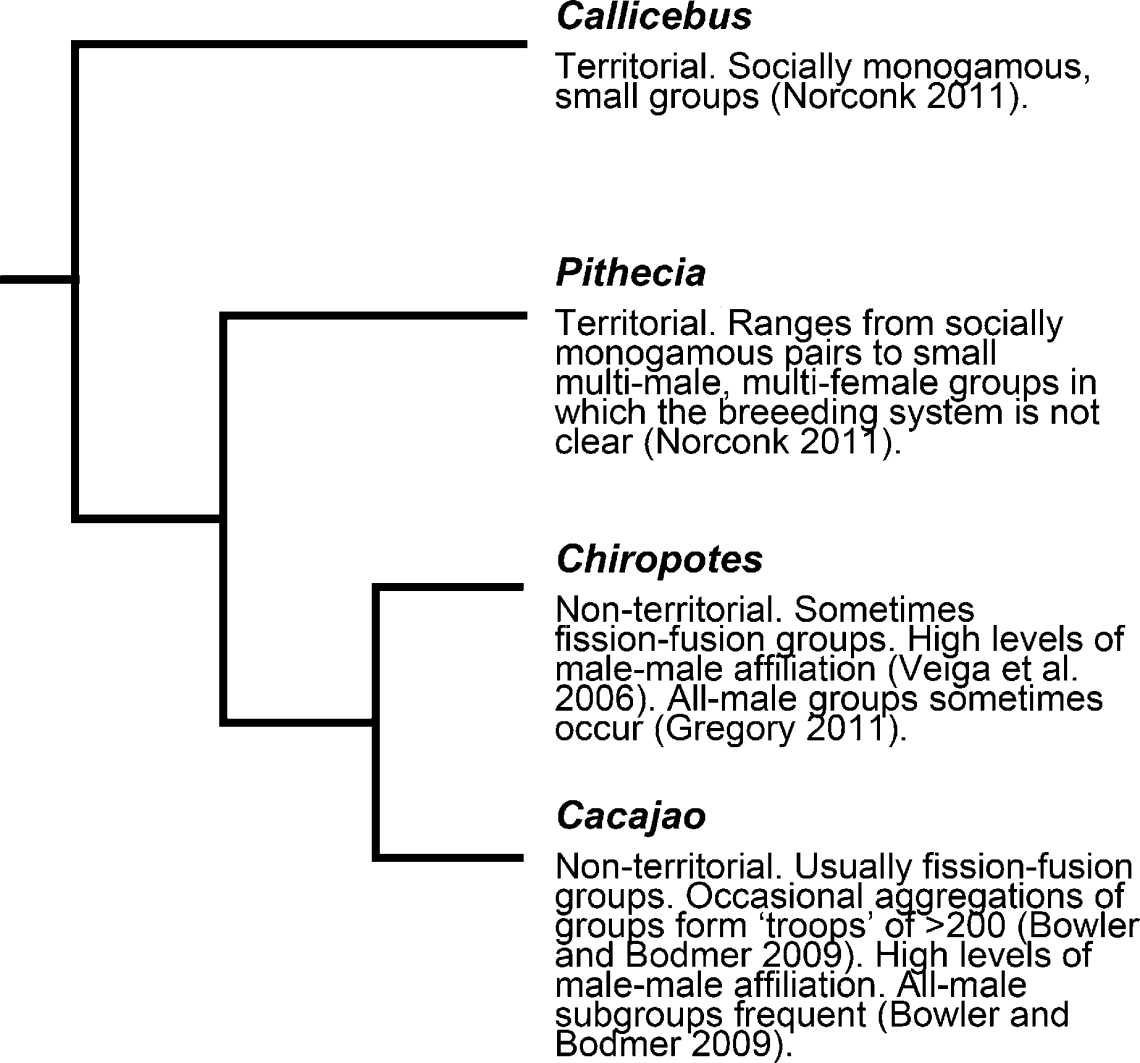
Phylogeny and social system in the Pitheciidae. Using phylogeny of Perelman *et al*. (2011).

As with other multilevel primate species, there are two alternative hypotheses for the origin of the social organization of *Cacajao*. Ancestrally solitary OMUs, or pairs, could have converged to form large groups, as assumed for *Rhinopithecus*, or they could have had an mm–mf organization that later became substructured. The social system of *Cacajao* is not well known, and similarly few studies have considered their social behavior. Regardless of whether *Cacajao* groups are organized into mm–mf or OMUs within larger groups, we can plot their social systems with those of other pitheciids on a phylogeny to determine the most likely evolutionary path of uakari social organization (Fig. 5). Titi monkeys (*Callicebus*) form territorial pairs, as do many groups of saki monkeys (*Pithecia*), and this is perhaps the ancestral pitheciid condition. *Pithecia* groups sometimes contain more than one male (Norconk 2011), providing evidence of the potential to deviate from monogamy and also of potential flexibility in social organization of the kind we are proposing for *Cacajao*, albeit at the other end of the scale for group size. *Chiropotes* is very closely related to *Cacajao*, having diverged relatively recently (*ca*. 6–7 million yr; Perelman *et al.* 2011). Chiropotes also occurs in large groups (>50 individuals; Norconk 2011), and although several groups studied have foraged as a single unit (Ayres 1989), low cohesion and fission–fusion have been shown in other populations (Silva and Ferrari 2009; Veiga and Silva 2005; Veiga *et al*. 2006), which might suggest a grouping pattern similar to that of *Cacajao*. We can hypothesize that the common ancestor of *Chiropotes* and *Cacajao* also lived in large groups that evolved through the association of ancestrally solitary OMUs or more likely from pairs similar to those seen in titi and saki monkeys.

## Future Directions

Details of the social organization of *Cacajao calvus* remain unresolved. Studies have failed to conclusively distinguish between an OMU or mm–mf breeding system, a third tier of social organization has yet to be confirmed, and the nature of m–m coalitions is unclear. Difficulties in observing the animals in the wild have been the major factor in restricting knowledge of this genus, but progress is being made at a number of sites that should allow these questions to be answered. *Cacajao* represents an interesting opportunity to test general theories on the evolution of OMUs, m–m affiliation, and coalition. Testing these hypotheses will require identifiable or marked uakaris, which will have to be followed, and their social interactions or proximity recorded over an extended period. To determine whether *Cacajao* has a two- or three-tiered society, the changing membership of groups and units will need to be tracked through time. Again this will require the reliable identification or marking of a number of individuals. If groups around the median size remain of consistent composition in the long term, especially following the fusioning and fissioning of groups to form ephemeral troops, then the three-tier concept would be confirmed.
